# Quantification of damage to plasmid DNA from 35 MeV electrons, 228 MeV protons and 300 kVp X-rays in varying hydroxyl radical scavenging environments

**DOI:** 10.1093/jrr/rrad032

**Published:** 2023-05-06

**Authors:** Hannah C Wanstall, Nicholas T Henthorn, James Jones, Elham Santina, Amy L Chadwick, Deepa Angal-Kalinin, Geoffrey Morris, John-William Warmenhoven, Rob Smith, Storm Mathisen, Michael J Merchant, Roger M Jones

**Affiliations:** Department of Physics and Astronomy, Faculty of Science and Engineering, The University of Manchester, Oxford Road, Manchester M13 9PL, UK; Manchester Academic Health Science Centre, The Christie NHS Foundation Trust, Wilmslow Road, Manchester M20 4BX, UK; The Cockcroft Institute, Daresbury Laboratory, Daresbury, Warrington WA4 4AD, UK; Manchester Academic Health Science Centre, The Christie NHS Foundation Trust, Wilmslow Road, Manchester M20 4BX, UK; Division of Cancer Sciences, Faculty of Biology, Medicine and Health, School of Medical Sciences, The University of Manchester, Oxford Road, Manchester M13 9PL, UK; The Cockcroft Institute, Daresbury Laboratory, Daresbury, Warrington WA4 4AD, UK; ASTeC, STFC Daresbury Laboratory, Daresbury, Warrington WA4 4AD, UK; Division of Cancer Sciences, Faculty of Biology, Medicine and Health, School of Medical Sciences, The University of Manchester, Oxford Road, Manchester M13 9PL, UK; Division of Cancer Sciences, Faculty of Biology, Medicine and Health, School of Medical Sciences, The University of Manchester, Oxford Road, Manchester M13 9PL, UK; The Cockcroft Institute, Daresbury Laboratory, Daresbury, Warrington WA4 4AD, UK; ASTeC, STFC Daresbury Laboratory, Daresbury, Warrington WA4 4AD, UK; The Cockcroft Institute, Daresbury Laboratory, Daresbury, Warrington WA4 4AD, UK; ASTeC, STFC Daresbury Laboratory, Daresbury, Warrington WA4 4AD, UK; Manchester Academic Health Science Centre, The Christie NHS Foundation Trust, Wilmslow Road, Manchester M20 4BX, UK; Division of Cancer Sciences, Faculty of Biology, Medicine and Health, School of Medical Sciences, The University of Manchester, Oxford Road, Manchester M13 9PL, UK; The Cockcroft Institute, Daresbury Laboratory, Daresbury, Warrington WA4 4AD, UK; ASTeC, STFC Daresbury Laboratory, Daresbury, Warrington WA4 4AD, UK; The Cockcroft Institute, Daresbury Laboratory, Daresbury, Warrington WA4 4AD, UK; ASTeC, STFC Daresbury Laboratory, Daresbury, Warrington WA4 4AD, UK; Manchester Academic Health Science Centre, The Christie NHS Foundation Trust, Wilmslow Road, Manchester M20 4BX, UK; Division of Cancer Sciences, Faculty of Biology, Medicine and Health, School of Medical Sciences, The University of Manchester, Oxford Road, Manchester M13 9PL, UK; Department of Physics and Astronomy, Faculty of Science and Engineering, The University of Manchester, Oxford Road, Manchester M13 9PL, UK; The Cockcroft Institute, Daresbury Laboratory, Daresbury, Warrington WA4 4AD, UK

**Keywords:** very high-energy electrons (VHEE), protons, relative biological effectiveness (RBE), DNA damage, hydroxyl radicals

## Abstract

The pBR322 plasmid DNA was irradiated with 35 MeV electrons, 228 MeV protons and 300 kVp X-rays to quantify DNA damage and make comparisons of DNA damage between radiation modalities. Plasmid was irradiated in a medium containing hydroxyl radical scavengers in varying concentrations. This altered the amount of indirect hydroxyl-mediated DNA damage, to create an environment that is more closely associated with a biological cell. We show that increasing hydroxyl scavenger concentration significantly reduced post-irradiation DNA damage to pBR322 plasmid DNA consistently and equally with three radiation modalities. At low scavenging capacities, irradiation with both 35 MeV electrons and 228 MeV protons resulted in increased DNA damage per dose compared with 300 kVp X-rays. We quantify both single-strand break (SSB) and double-strand break (DSB) induction between the modalities as a ratio of yields relative to X-rays, referred to as relative biological effectiveness (RBE). RBE_SSB_ values of 1.16 ± 0.15 and 1.18 ± 0.08 were calculated for protons and electrons, respectively, in a low hydroxyl scavenging environment containing 1 mM Tris–HCl for SSB induction. In higher hydroxyl scavenging capacity environments (above 1.1 × 10^6^ s^−1^), no significant differences in DNA damage induction were found between radiation modalities when using SSB induction as a measure of RBE. Considering DSB induction, significant differences were only found between X-rays and 35 MeV electrons, with an RBE_DSB_ of 1.72 ± 0.91 for 35 MeV electrons, indicating that electrons result in significantly more SSBs and DSBs per unit of dose than 300 kVp X-rays.

## INTRODUCTION

A number of external beam radiotherapy modalities are used for cancer treatment, including X-rays, electrons, protons and heavy ions. In all modalities, the radiation dose is used as a physical measure in place of biological effectiveness. However, tumour control and the risk of normal tissue complication are not well described by dose when comparing across modalities.

Very high-energy electrons (VHEEs), generally defined as 50–250 MeV electrons, are a potential future radiotherapy treatment. VHEE has been proposed as a new technology for cancer treatment due to several physical characteristics that could potentially offer advantages for some clinical cases. The possibility of using VHEE for cancer treatment was first described by DesRosiers *et al.* [1], who showed that electrons over 150 MeV are capable of reaching deep-seated tumour sites in patients using simulations in water. Increasing electron energy significantly reduces scattering and results in a smaller penumbra, which reduces the dose to lateral healthy structures in patients. VHEE has the potential to revolutionize the treatment of specific cancer types, especially those located in inhomogeneous regions such as lung and prostate cancers [2]. Practically, a future VHEE machine could potentially offer a cheaper solution to proton and heavy ion therapy machines due to major technological advancements such as high-gradient accelerating structures [3, 4]. The Compact Linear Collider (CLIC)-like technology [5] could theoretically produce a compact VHEE machine that could reasonably fit within a clinical setting, without the need for large multi-story systems, as with clinical proton machines. VHEE also has the potential to be delivered at high dose rates, meaning that the implementation of FLASH radiotherapy is also considered a potential future application and benefit.

Proton radiotherapy is gaining popularity worldwide as a cancer treatment, particularly in the case of paediatric, head and neck, brain and breast cancers [6]. The modality is suited to tumours close to sensitive organs due to the sharp dose falloff distal to the depth of maximum dose (Bragg peak) [7], in contrast to X-ray therapy where dose is deposited across the full depth. The depth of the Bragg peak is tuned by proton energy, with higher energy protons travelling further. Several Bragg peaks are combined in therapy, referred to as a spread-out Bragg peak, to ensure that a target tumour is covered fully while maintaining a low dose to healthy tissue [8].

To compare across different radiation modalities, a relative biological effectiveness (RBE) value is taken into account. RBE is currently used clinically for protons and heavy ions. RBE is defined as a ratio of two doses, where test radiation is compared to a reference modality and results in the same amount of biological damage [9], effectively scaling the dose to match the biological outcome. An RBE value should be quantified before new radiation delivery technology can be used safely and precisely in the clinic. Accurate values for RBE specific to physical parameters such as particle type, beam energy and linear energy transfer (LET) could potentially aid more optimal and therefore safer treatment plans [10]. Biological parameters such as cell type, tumour environment and biological endpoint should also be explored, as these are known to affect RBE. For proton therapy, a constant RBE of 1.1 is applied, chosen as a conservative value that does not result in significant underdosing or overdosing of the tumour volume. The situation is more complicated in carbon therapy, where, due to a significantly higher LET, the RBE effects are more pronounced. Here, models of variable RBE are applied at the treatment planning stage [11, 12].

RBE values for 100–200 MeV electrons have been investigated previously in our Manchester MEW research group by Small *et al.* via plasmid irradiation studies using the CLEAR beamline at CERN [13]. Results from this study indicate that VHEE has an RBE value of between 1.09 and 1.19, meaning that ~10–20% more DNA damage occurs per dose of VHEE compared with X-ray irradiation. A limitation of Small *et al.*’s study is that DNA irradiations were performed in low hydroxyl scavenging capacity conditions (1 mM Tris–HCl) or a scavenging capacity of ~1.1 × 10^6^ s^−1^ [14]). Low scavenging conditions are not optimal for representing how VHEE reacts within a cellular environment, as cellular scavenging capacity is ~3.0 × 10^8^ s^−1^ [15].

A static RBE value of 1.1 is currently used clinically for proton treatment planning; however, *in vitro* literature data suggest that RBE is variable, reaching a maximum at the Bragg peak [16]. Paganetti *et al.* indicated that proton RBE changes significantly over the distance of the proton Bragg peak—from 1.1 at the entrance region to 1.7 at the distal falloff [9], due to increasing LET. Proton RBE has also been shown to have a dependency on cell type and biological endpoint [9]. RBE has been shown to vary significantly with LET in the case of protons. The measurement of RBE_SSB_ and RBE_DSB_ is critical for understanding the physical effects of X-rays, protons and electrons. This work investigates these effects by comparing three low LET radiations of each radiation type to determine differences between modalities.

The main aim of this work is to determine accurate RBE values of DNA damage induction for both 35 MeV electrons and 228 MeV protons, in comparison to conventional X-rays. In this study, pBR322 plasmids were irradiated, and the resulting DNA damage was quantified by measuring the proportions of supercoiled (SC), open circular (OC) and linear (L) plasmid structures. SC structure represents undamaged plasmid, still in its native state. OC and L represent plasmid structures after they have a single-strand break (SSB) or a double-strand break (DSB) [17]. These three structures can be separated using gel electrophoresis, allowing the proportion of each structure to be quantified [18].

Hydroxyl radical scavengers were added to the pBR322 plasmid DNA solution to create scavenging capacity environments with up to 3.3× more hydroxyl scavenging ability than a cellular environment. The increased scavenging capacity aids in creating a more accurate representation of a cellular environment by reducing the number of available hydroxyl radicals. Investigation of DNA damage across a range of chemical environments provides us with important information on how radiation modalities react with their aqueous environments to produce reactive oxygen species. Production of these species is ultimately how the majority of radiation-mediated DNA damage occurs, meaning the measurement of chemical interactions is vital for the validation of mechanistic RBE models. This also allows for the investigation of direct vs indirect (radical mediated) effects of DNA damage between modalities. Hydroxyl radicals play a major part in creating radiation-induced DNA damage via indirect mechanisms [5]. Tris and DMSO have been used as hydroxyl radical scavengers in this study [6, 7].

## MATERIALS AND METHODS

### Preparation of samples

Samples of 100 μg/ml pBR322 plasmid DNA (New England Bioscience) were prepared in 1.5 ml Eppendorf tubes with varying concentrations of scavengers Tris (Thermo Fisher Scientific, AN9855G) or DMSO (Sigma Aldrich, D8418). Each sample was prepared to a total volume of 8 μl (for electrons and X-rays) or 16 μl (for protons) using stock pBR322 plasmid DNA (1000 μg/ml), diluted with distilled water, and either 1 M Tris or 1 M DMSO. Samples were irradiated within 1.5 ml Eppendorf tubes for X-rays and electrons. Samples were irradiated in 96-well plates (Corning, 3596) for protons. The use of 96-well plates during proton irradiations was due to the horizontal beam source—samples had to be presented to the beam using a robot and therefore had to be placed in a vessel of suitable dimensions. The following concentrations of Tris were used: 1, 10, 100 and 900 mM, and the following concentrations of DMSO were used: 0.1, 1, 10 and 100 mM. The pBR322 plasmid supplied by New England Biosciences is suspended in a buffer containing 10 mM Tris, meaning that all samples have a residual concentration of Tris–HCl hydroxyl scavenger (1 mM).

### Irradiation of pBR322

The plasmid samples were irradiated across three research centres—electron irradiations took place at STFC Compact Linear Accelerator for Research and Applications (CLARAs) facility at Daresbury Laboratory, UK [19]. Proton irradiations took place in The University of Manchester proton research room at The Christie Hospital, Manchester, UK. X-ray irradiations were performed using the Xstrahl CIX3 Cell Irradiator in the Oglesby Cancer Research Building (OCRB), Manchester, UK. Samples of each scavenger concentration were then irradiated at five doses, alongside an unirradiated control. Irradiations were completed using 300 kVp X-rays to represent a photon control. The experiment for each individual radiation modality was completed three times. For comparison, the experiment was also performed with 35 MeV electrons and 228 MeV protons. Doses of 15, 30, 45, 60 and 75 Gy were used to irradiate samples of each scavenger concentration. It should be noted that although these doses were consistent for X-ray and proton samples, the doses differed significantly for 35 MeV electron irradiations, due to the varying charge supplied by CLARA, as well as the positioning of the sample within the Gaussian beam spot. Delivered doses were validated using Monte Carlo simulations alongside EBT-XD GafChromic film.

### X-ray experimental setup on Xstrahl CIX3 cell irradiator at OCRB

pBR322 samples were irradiated with 300 kVp X-rays using the Xstrahl CIX3 Cell Irradiator at the OCRB. To irradiate, Eppendorf tubes were laid flat on a turntable within the irradiator, due to the X-ray source being vertical. The turntable ensured uniform irradiation and mitigated dose hot/cold spots. The dose was controlled by the X-ray source based on time, with pre-calibration performed. Post-irradiation, all plasmid samples were then analysed in the OCRB through continuous field gel electrophoresis.

### Electron experimental setup at the CLARAs facility at Daresbury Laboratory

**Fig. 1:**
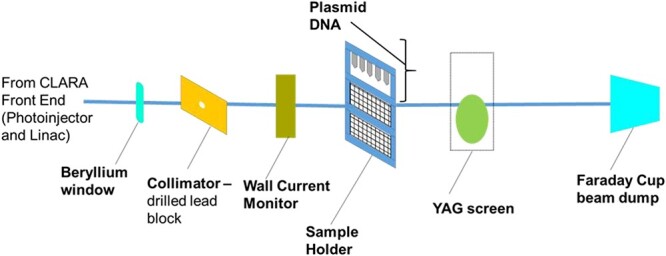
A schematic representation of the experimental beamline on CLARA front end. Electrons from the photocathode are accelerated by S-band linacs, steered by dipole magnets and focussed by quadrupole magnets. The accelerator ends at the Beryllium window with electrons transported thereafter through ~30 cm in air to the experimental beamline, terminating in the Faraday cup for charge measurement.

pBR332 samples were prepared at the OCRB and then transported to and from Daresbury Laboratory on ice to maintain sample stability. At Daresbury Laboratory (Fig. 1), samples were slotted into the custom-made sample holder in front of the beamline. This allowed samples to be irradiated in batches of eight, with the samples moved in front of the beam by a robotic stage. The 35 MeV electron beam was collimated by a drilled lead block to minimize the background due to dark current. Beam current was measured by a wall current monitor installed close to the sample, and the yttrium aluminium garnet screen was used to aid in beam alignment. Dose was controlled by measuring the beam current on a wall current monitor. Dose was predicted using Monte Carlo simulation, with EBT-XD GafChromic film used as a secondary check. Post-irradiation, plasmid samples were analysed at the OCRB.

### Proton experimental setup at the Christie proton centre research beamline

**Fig. 2:**
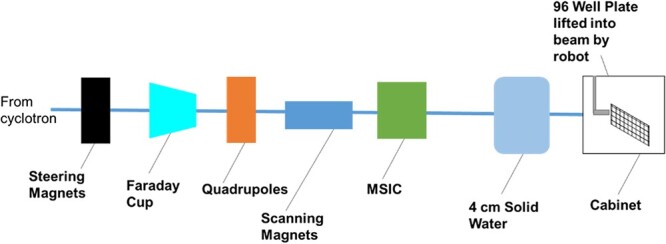
A schematic representation of the experimental area of the Proton Research Beamline at The Christie Hospital. Protons are accelerated with the cyclotron, focussed by the quadrupoles, scanned horizontally and vertically by the magnets and dosimetrically scored by the MSIC. The plasmid DNA sample is housed in the cabinet and presented to the beam by a six-axis robotic arm.

pBR322 samples were prepared at the OCRB. Samples were then transported to and from The Christie Hospital on ice to maintain sample stability. Irradiation of the 96-well plates took place inside a cabinet with samples presented to the spot-scanning proton beam (Fig. 2) by a six-axis robotic arm. The sample was irradiated with a 60 × 60 mm field, with 2.5 mm proton spot spacing. The dose was controlled by a multi strip ionization chamber (MSIC) and verified with a PTW microdiamond chamber. The irradiation used 245 MeV protons with 4 cm of solid water before the sample (sample energy calculated as 228 MeV by Geant4 Monte Carlo simulation [20–22]). Post-irradiation, plasmid samples were analysed at the OCRB.

### Dosimetry

X-ray and proton dosimetry measurements were pre-calibrated, with specific errors on dose recorded in Table 1. These calibrations do not exist for the CLARA facility and were instead calculated using TOPAS Monte Carlo simulation [23], with validation through EBT-XD GafChromic film. This method has been previously validated to have a maximum error of ~5% for electron energies between 60 and 205 MeV [2, 24]. Comparisons between TOPAS Monte Carlo simulation and the specific batch of EBT-XD films used resulted in a dose error of 5.68% in this case.

**Table 1:** Basic beam parameters for each radiation modality.

Radiation modality	Energy (MeV)	LET (keV/μm)	Average dose rate (Gy/s)	Dose error (SD%)
X-ray	0.3 (max)	0.20	0.04	1.34
Electron	35	0.21	3.08	5.68
Proton	228	0.42	0.12	0.63

LET values for X-rays and protons were simulated using Geant-4 DNA, where particles were simulated across a volume of water to determine the average energy loss for primary particles [25–27]. LET values for electrons were taken from ESTAR [28]. The average dose rates were calculated using a combination of time data from irradiations and dose to the sample, where the total dose to the sample was divided by the total time taken for the irradiation. X-ray and proton dose error were calculated from previous quality assurance dosimetry measurements. EBT-XD film has been shown to have 0.5–5% error by the manufacturers [23, 29].

During electron experiments, EBT-XD GafChromic film was placed behind the samples to record the dose in this area, after the electrons had passed through the Eppendorf tube. The dose lost through the Eppendorf tube and sample was then simulated in TOPAS to create a value for the dose hitting the front of the tube containing the sample. This was also verified through extrapolation of the Gaussian profile of the beam, based on the profile recorded on EBT-XD film. The overall set up was simulated in TOPAS using the charges and beam profiles recorded during the experimental run. There was a difference of 5.68% between the doses recorded by EBT-XD and TOPAS simulation.

The doses for each radiation modality can therefore be defined as the dose that hits the front of the Eppendorf tube, or plate, in the location of the sample. Although the radiation has to then move through the wall of the tube (~ 1 mm polypropylene), this effect was counted as negligible due to the change in dose having minimal difference between modalities (>0.5%).

The beam characteristic parameters for each radiation modality are indicated in Table 1. There were variations between irradiation methods between modalities that reflect the differences. Electron irradiations consisted of a single spot (σ = 2.5 ± 0.07 mm), aligned with the sample at the bottom of the Eppendorf tube. A typical bunch charge during the experiment was 45–70 pC and this was administered at a rate of 10 Hz. X-ray samples were irradiated in the Xstrahl CIX3 Cell Irradiator—this device generates X-rays at a constant energy and dose rate in a shaped radiation beam over the sample. During proton irradiations, a pre-calibrated spot scanning method of overlapping beam spots was used to cover the entire plate uniformly. The average dose rates for these have been described in Table 1; however, it should be noted that there will be unavoidable differences in the structure of irradiations between modalities.

### Analysis of DNA by electrophoresis gel

When plasmid DNA is irradiated, DNA damage can occur in the form of SSBs and DSBs. To quantify the amount of damage to the plasmid, the structure of plasmid DNA in each sample was measured by agarose gel electrophoresis. The native structure of the plasmid is in its SC form; however, when there is a SSB in the plasmid structure, the plasmid relaxes to become OC. When there is an occurrence of a DSB, the plasmid structure then becomes L.

About 1% w/v agarose gel was prepared using 1X TAE buffer (Thermo Fisher Scientific, B49), 1% w/v agarose (Sigma Aldrich, A9539) and 1X SYBR Safe DNA gel stain (Thermo Fisher Scientific, S33102). Once the gel was set, it was submerged in 0.5× TAE solution, and 5 μl samples were loaded into the wells of the electrophoresis gel, containing 83.3 μg/ml pBR322 plasmid, 1X loading dye (Thermo Fisher Scientific, R0611) and relevant scavenger concentrations. About 100 V were then applied to the gel for 2 h, and the gels were imaged using a ChemiDoc MP UV imager (BioRad). All images were taken on the ChemiDoc ‘SYBR Safe’ setting, and the automatic ‘Rapid Exposure’ setting was used to take all images.

Images were then analysed using Fiji software. To do so, the integrated density of each band was measured to quantify the amount of DNA in each form. The integrated densities of each band were then used to calculate the relative proportion of SC, OC and L plasmids for each sample.

### Analysis of pBR322 structure using the McMahon fit

The proportion of plasmid form was converted into SSB and DSB yields according to McMahon and Currell [30]. The McMahon curves were fitted separately for each scavenger concentration and repeat to allow a value for SSB and DSB frequency to be determined. To do so, relative proportions of SC, OC and L plasmids are fitted to the following equations:


(1) \begin{equation*} S={S}_0{e}^{-\left({\beta}_s+{\beta}_D\right)D} \end{equation*}

(2) \begin{equation*} C={e}^{-{\beta}_DD}\left[{C}_0{e}^{-\frac{1}{2}{\beta}_s^2\rho{D}^2}+{S}_0\left({e}^{-\frac{1}{2}{\beta}_s^2\rho{D}^2}-{e}^{-{\beta}_sD}\right)\right] \end{equation*}

(3) \begin{equation*} L=1-\left({C}_0+{S}_0\right){e}^{-\left({\beta}_DD+\frac{1}{2}{\beta}_s^2\rho{D}^2\right)} \end{equation*}

where *S*, *C* and *L* are the proportions of SC, OC and L plasmid structures, respectively. ${S}_0$, ${C}_0$ and ${L}_0$ are the proportions of these structures in an unirradiated control. *D* is the dose in Gy. $\rho$ is the ratio between the length of the plasmid and the maximum distance between SSBs that could result in a DSB (taken as 10 base pairs). ${\beta}_s$ and ${\beta}_D$ are the frequencies of SSBs and DSBs per unit dose and are fit across the measured experimental dose range. Microsoft Excel solver (2016) was used to create nonlinear fits to these three equations simultaneously, to find values of ${\beta}_s$ and ${\beta}_D$, while minimizing error of the fit.

### Statistical analysis

A two-way analysis of variance (ANOVA) was applied to the full dataset to detect statistical differences between radiation modalities and scavenger concentrations. This was followed up by Tukey’s multiple comparison test to show statistical differences between 300 kVp X-rays and either 35 MeV electrons or 228 MeV protons, for each scavenger concentration. *P*-values <0.05 were considered to be statistically significant. All values are presented as mean values ± one standard deviation (SD).

### Calculation of scavenging capacities

Samples were prepared to have specific scavenger concentrations as indicated in Table 2. The pBR322 plasmid arrived diluted in 10 mM Tris–HCl and 1 mM EDTA. Where samples are suspended alongside varying concentrations of Tris buffer, this residual amount of Tris–HCl has been accounted for. It should be noted that where samples are suspended in varying concentrations of DMSO, there is residual 1 mM Tris–HCl hydroxyl scavenger and 0.1 mM EDTA alongside the DMSO concentration noted.

**Table 2:** Hydroxyl radical scavenging capacities for concentrations of Tris and DMSO (+ residual Tris–HCl)

Scavenger constituents	Scavenging capacity (s^−1^)	Scavenging capacity/cellular scavenging capacity
1 mM Tris–HCl	1.1 × 10^6^	0.004
10 mM Tris	1.1 × 10^7^	0.037
100 mM Tris	1.1 × 10^8^	0.370
900 mM Tris	9.9 × 10^8^	3.300
0.1 mM DMSO (+ 1 mM Tris–HCl)	1.8 × 10^6^	0.006
1 mM DMSO (+ 1 mM Tris–HCl)	7.7 × 10^6^	0.026
10 mM DMSO (+ 1 mM Tris–HCl)	6.7 × 10^7^	0.222
100 mM DMSO (+ 1 mM Tris–HCl)	6.6 × 10^8^	2.200

The scavenging capacities for each concentration are also compared with cellular scavenging capacities (taken as 3.0 × 10^8^ s^−1^ [15]). Values are calculated assuming a scavenging capacity of 1.1 × 10^9^ M^−1^ s^−1^ for both pure Tris [14] and Tris–HCl, and 6.6 × 10^9^ M^−1^ s^−1^ for DMSO [31]. Residual EDTA buffer has not been considered during these calculations because the primary use of EDTA is to deactivate DNase and RNase enzymes, rather than acting as a radical scavenger.

## RESULTS

**Fig. 3:**
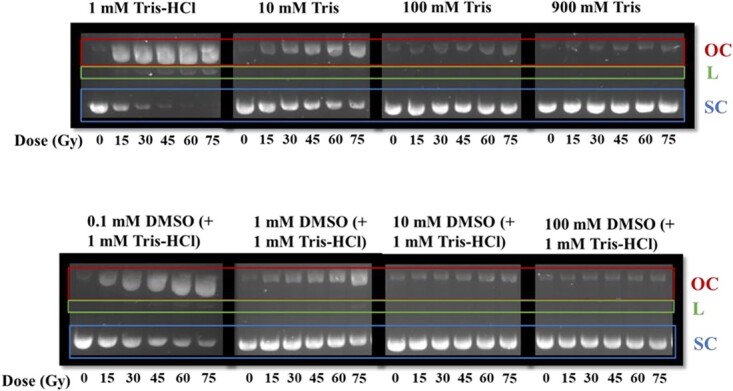
Image from agarose gels of X-ray irradiated samples where bands on the gel represent the pBR322 plasmid DNA. Plasmid DNA is separated out into three structures, SC (blue—bottom), OC (red—top) and L (green—middle), which are indicated by the coloured box surrounding the bands, and neighbouring labels. Image is original image as taken, with no post-processing. The dose (Gy) is indicated by *x*-axis labelling of each band below image, and the scavenger concentration is indicated by labelling above each box.

The pBR322 plasmid DNA was irradiated at six dose points up to 75 Gy (including an unirradiated sample). The pBR322 plasmid DNA structures were then separated into SC, OC and L via agarose gel electrophoresis, as previously described. An example of the agarose gel electrophoresis is shown in Fig. 3 for X-ray irradiated samples (representative of one experimental repeat). Figure 3 shows that the majority of unirradiated pBR322 plasmid remains in its SC structure (0.85 ± 0.09) with a small amount of the plasmid in OC structure (0.15 ± 0.09), due to a proportion of the plasmids containing residual SSBs caused by storage and handling. The values stated represent the mean proportions of plasmid structure across all modalities and scavenger concentrations ± SD. No plasmid can be observed in the L structure when unirradiated, showing that no DSBs are induced without irradiation. This unirradiated control remains similar in each condition including with increasing scavenger concentration.

Figure 3 shows that for the lower scavenger concentrations (1 mM Tris–HCl, 10 mM Tris, 0.1 mM DMSO and 1 mM DMSO), the intensity of the OC band increases, and the SC band decreases visibly with increasing dose. The lowest scavenger concentrations (1 mM Tris–HCl and 0.1 mM DMSO) also have visible evidence of L structure plasmid when irradiated. This effect can be visualized best with the 1 mM Tris–HCl condition, where the intensity of the L structure band can be observed to increase significantly with dose. In conditions containing higher scavenger concentrations, the effect of dose on plasmid structure is significantly reduced. The linear bands cannot be seen at these high concentrations, indicating no, or an undetectable number of DSBs. At the highest scavenger concentrations, the majority of the plasmid structure remains in its SC form, similar to an unirradiated control, even at high doses (up to 75 Gy).

**Fig. 4:**
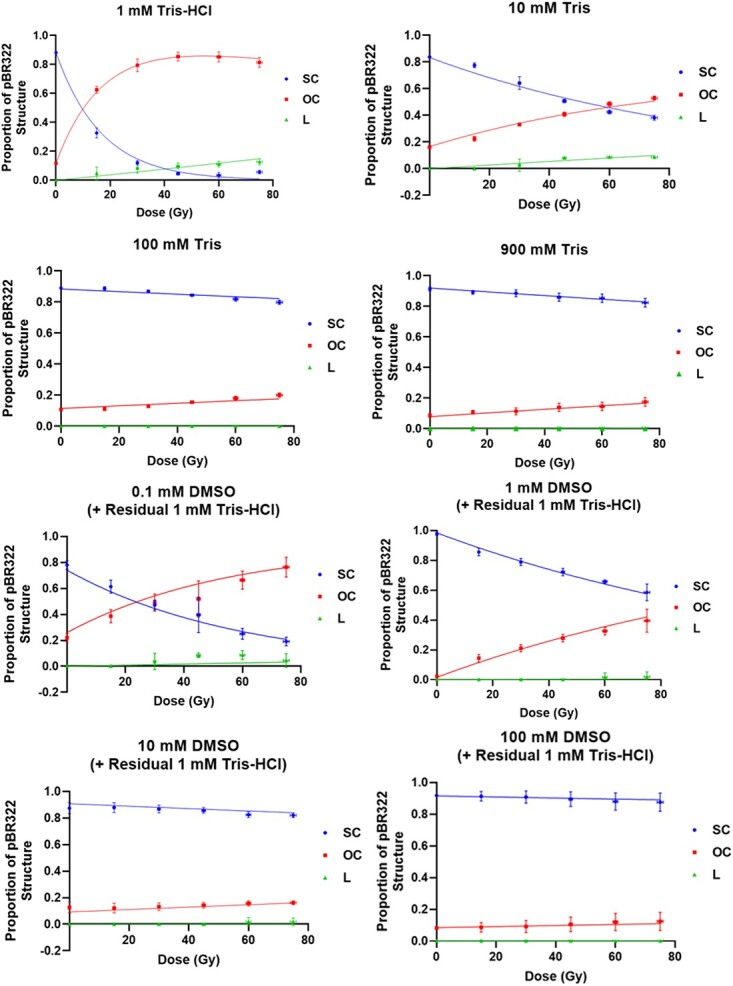
Proportions of SC, OC and L pBR322 plasmid as a function of dose, for various scavenger concentrations of Tris (1, 10, 100 and 900 mM) and DMSO (+ residual 1 mM Tris) (0.1, 1, 10 and 100 mM). All graphs are taken from irradiation with X-rays, where points are the mean from three statistical repeats (± 1 SD). Fits represent McMahon curves for one statistical repeat. *X*-axis error bars for dose (1.34%) have been added but are too small to be visible in several of the data points.

The integrated density of each band was extracted from agarose gel images and then converted into a relative proportion of plasmid structure. The McMahon model (Equations 1–3) was fitted to these structure proportions to produce curves that represent the proportion of SC, OC and L plasmids. Individual McMahon curves were completed for each of the three experimental repeats, due to the varying structure proportions of the unirradiated control for each repeat and condition. Curves were produced for each individual condition (for each scavenger concentration and radiation modality). An example of these curves for one statistical repeat of X-ray samples is shown in Fig. 4. Data points are mean values of three statistical repeats.

**Fig. 5:**
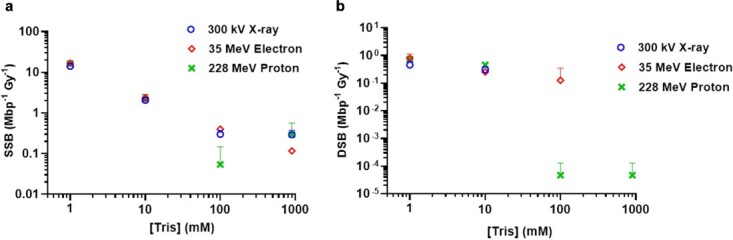
The number of SSBs (**a**) and DSBs (Mbp^−1^ Gy^−1^) (**b**) induced in response to changing Tris concentration. The varying modalities are shown by different coloured points. Error bars represent SD across three irradiation repeats.

**Fig. 6:**
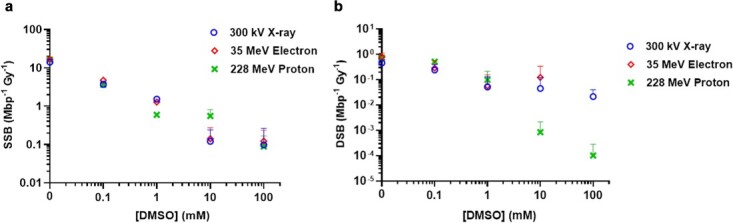
The number of SSBs (**a**) and DSBs (Mbp^−1^ Gy^−1^) (**b**) induced in response to changing DMSO concentration. The varying modalities are shown by different coloured points. Error bars represent SD across three irradiation repeats.

McMahon curves have been fit for three repeats of each modality: 300 kVp X-rays, 35 MeV electrons and 228 MeV protons. Each fit produces a value for SSB and DSB induction. These values were then averaged between the three repeats to allow comparisons between radiation modalities, at each scavenger concentration. These results are shown in Figs 5 and 6.

All the three radiation modalities result in decreased DNA damage with increasing scavenger concentration, which is consistent with conventional X-rays (Figs 3 and 4). Figure 5 indicates the damage caused by increasing the concentration of hydroxyl radical, Tris. This is measured by SSB and DSB induction per dose (Fig. 5a and b).

Figure 6 indicates the damage caused by increasing the concentration of another hydroxyl radical, DMSO. This is measured by SSB and DSB induction per dose (Fig. 6a and b).

Figures 5 and 6 show that increasing scavenger concentration results in a reduction of DNA damage. Variability between the three repeats increases at higher scavenging capacity due to the low rates of damage induction. This results in large differences between the modalities.

At high scavenger concentrations, DSB induction is a rare occurrence, meaning that there is higher variability between repeats than with SSB induction. It is evident that increasing scavenging capacity results in less DNA damage overall for each modality; however, there are differences in the quantity of DNA damage occurring at specific scavenger concentrations. This is summarized in Table 3, which highlights the number of SSB (Mbp^−1^ Gy^−1^) and induced by each radiation modality at each hydroxyl scavenger concentration, as well as the significant differences identified in SSB and DSB induction, between radiation modalities.

**Table 3:** The frequency of SSBs and DSBs (Mbp^−1^ Gy^−1^) that are created as a result of irradiation with 300 kVp X-rays, 35 MeV electrons and 228 MeV protons at various concentrations of Tris and DMSO scavenger concentrations

Scavenger constituents	SSB (Mbp^−1^ Gy^−1^) ± SD			Statistical result (based on two-way ANOVA)
	300 kVp X-ray	35 MeV electrons	228 MeV protons	
1 mM Tris–HCl	14.08 ± 0.81	16.68 ± 0.68	16.40 ± 1.89	**Significant between modalities:** 300 kV X-ray vs 35 MeV electron (*P* = 0.0003) 300 kV X-ray vs 228 MeV proton (*P* = 0.0009)
10 mM Tris	2.07 ± 0.21	2.32 ± 0.45	2.15 ± 0.72	**n/s between modalities**
100 mM Tris	0.30 ± 0.07	0.40 ± 0.02	0.16 ± 0.09	**n/s between modalities**
900 mM Tris	0.29 ± 0.08	0.12 ± 0.01	0.30 ± 0.27	**n/s between modalities**
0.1 mM DMSO (+ 1 mM Tris–HCl)	3.70 ± 0.82	4.76 ± 0.31	3.64 ± 0.16	**n/s between modalities**
1 mM DMSO (+ 1 mM Tris–HCl)	1.53 ± 0.14	1.283 ± 0.22	0.59 ± 0.09	**n/s between modalities**
10 mM DMSO (+ 1 mM Tris–HCl)	0.12 ± 0.12	0.14 ± 0.13	0.55 ± 0.26	**n/s between modalities**
100 mM DMSO (+ 1 mM Tris–HCl)	0.10 ± 0.17	0.12 ± 0.11	0.09 ± 0.08	**n/s between modalities**
	DSB (Mbp ^−1^ Gy^−1^) ± SD			
1 mM Tris–HCl	0.46 ± 0.14	0.79 ± 0.34	0.73 ± 0.19	**Significant between modalities:** 300 kV X-ray vs 35 MeV electron (*P* = 0.0187) **n/s between modalities:** 300 kV X-ray vs 228 MeV proton
10 mM Tris	0.31 ± 0.04	0.26 ± 0.03	0.46 ± 0.07	**n/s between modalities**
100 mM Tris	0.00 ± 0.00	0.13 ± 0.22	4.63 × 10^−5^ ± 8.13 × 10^−5^	**n/s between modalities**
900 mM Tris	0.00 ± 0.00	0.00 ± 0.00	4.63 × 10^−5^ ± 8.13 × 10^−5^	**n/s between modalities**
0.1 mM DMSO (+ 1 mM Tris–HCl)	0.24 ± 0.16	0.29 ± 0.26	0.52 ± 0.06	**n/s between modalities**
1 mM DMSO (+ 1 mM Tris–HCl)	0.05 ± 0.08	0.06 ± 0.10	0.10 ± 0.11	**n/s between modalities**
10 mM DMSO (+ 1 mM Tris–HCl)	0.05 ± 0.08	0.12 ± 0.21	8.47 x 10^−4^ ± 1.35 × 10^−3^	**n/s between modalities**
100 mM DMSO (+ 1 mM Tris–HCl)	0.02 ± 0.02	0.00 ± 0.00	1.02 x 10^−4^ ± 1.77 × 10^−4^	**n/s between modalities**

SD represents the standard deviation across three radiation repeats. n/s = not significant. Where the statistical result is stated as n/s (not significant), the two-way ANOVA test did not find any statistical difference in SSB, or DSB frequency between protons, electrons or X-rays.

The majority of the hydroxyl scavenger concentration conditions did not show any significant differences in DNA damage between radiation modalities, based on three irradiation repeats. Only the condition with the lowest scavenger concentration, and therefore with the most DNA damage occurring, resulted in a significant difference between radiation modalities. The 1 mM Tris–HCl condition resulted in both 35 MeV electrons and 228 MeV protons producing higher levels of DNA damage in comparison to 300 kVp X-rays. X-rays were found to induce 14.08 ± 0.81 SSB’s (Mbp^−1^ Gy^−1^), with electrons and protons inducing 16.68 ± 0.68 and 16.40 ± 1.89 SSBs (Mbp^−1^ Gy^−1^), respectively. This indicates that when considering only DNA damage by SSBs, both these modalities have a higher RBE_SSB_ than conventional X-rays. About 35 MeV electrons resulted in more SSBs (Mbp^−1^ Gy^−1^) than protons; however, this difference was not found to be significant.

At 1 mM Tris–HCl, DSB induction was also higher for 35 MeV electrons and 228 MeV protons in comparison to 300 kVp X-rays; however, this was only significant in the case of 35 MeV electrons. About 300 kVp X-rays were shown to induce 0.46 ± 0.14 DSBs (Mbp^−1^ Gy^−1^), whereas 35 MeV electrons induced 0.79 ± 0.34 DSBs (Mbp^−1^ Gy^−1^). When considering the measure of RBE to be the induction of SSBs (Mbp^−1^ Gy^−1^) in an environment of 1 mM Tris–HCl, the RBE_SSB_ values for 228 MeV protons and 35 MeV electrons are calculated to be 1.16 ± 0.15 and 1.18 ± 0.08, respectively, where the error is SD across three irradiation repeats. When DSBs are considered as the measure of RBE, 35 MeV electrons have an RBE_DSB_ value of 1.72 ± 0.91, where the error is SD across three irradiation repeats.

## DISCUSSION

This work investigates the effect of radiation modality, as well as hydroxyl scavenger concentration on DNA damage to the pBR322 plasmid DNA. Our results show that 35 MeV electrons and 228 MeV protons have RBE_SSB_ values of 1.18 ± 0.08 and 1.16 ± 0.15, respectively, at low scavenging capacity (1 mM Tris–HCl only). At this low scavenging capacity, there was a significant difference between the amount of SSBs induced by both electrons and protons when compared with conventional X-rays. Our result also show that 35 MeV electrons have a significantly higher induction of DSBs in comparison to 300 kVp X-rays, with an RBE_DSB_ value of 1.72 ± 0.91. This result has a high error across three repeats, suggesting that RBE_SSB_ is a more precise measure than RBE_DSB_ in this study. No statistical differences between 300 kVp X-rays and 228 MeV protons were found when using DSB induction as the measure of RBE.

At higher scavenger concentrations, no significant differences were detected when comparing proton and electron results with conventional X-rays. This could indicate that protons and electrons only maintain their RBE above 1 when there are higher levels of DNA damage as a result of indirect hydroxyl-mediated damage. This could suggest that protons and electrons result in more damage than X-rays due to their increased ability to create hydroxyl radicals that can go on to attack the DNA structure. There are limitations to this finding, the main one being that although the Tris/DMSO environment does represent a cellular one more accurately than pure water experiments that are often reported in the literature. A water environment does not represent the true complexity of a cellular environment. The addition of a hydroxyl scavenger is a simple method of modelling the scavenging ability of the cell; however, it does not model how the various radical concentrations are maintained *in vivo* using intricate systems. The findings of this study, therefore, act as a first step and require validation through *in vitro* and *in vivo* experiment before the effects of radiation modality, in particular for high-energy electron or VHEE, can be fully understood.

As hydroxyl scavenger concentrations increase, we show that significantly less DNA damage takes place, to the point that no detectable DSBs occur, and the number of SSBs is negligible at 75 Gy for all modalities. This is expected due to the reduction in indirect hydroxyl-mediated damage and validates findings by Small *et al.* [13]. When indirect damage is significantly reduced by the addition of hydroxyl scavengers, there are no significant differences between the amounts of DNA damage induced. This suggests that electrons, protons and X-rays do not differ in the amount of SSBs or DSBs they induce by direct DNA damage per dose. This is a limitation within this study, as the accuracy of this technique is significantly compromised when DNA damage is at low levels. As a result, differences in modality cannot be reliably measured at high scavenger concentrations and values have such large error values, that if small differences occur between modalities, their detection would be unlikely in this dose range (up to 75 Gy). If there were capabilities to reach higher doses, this effect could potentially have been explored; however, the speed at which proton and X-ray samples could be irradiated was the limiting factor. Studies by Small *et al.* and Vysin *et al.* show this effect by irradiating dry pBR322 plasmid samples up to 6 and 2 kGy, respectively [32, 33]. This magnitude of dose (>1 kGy) is necessary to effectively measure changes in direct damage for similar protocols.

Small *et al.* [13] found a value of VHEE RBE_DSB_ of between ~1.1 and 1.2 for plasmids suspended in water when using DSB number as a measure of RBE. This is consistent with our own findings for 35 MeV electrons when using both DSB and SSB induction as our RBE indicator. This could suggest that electron RBE is energy independent, with energies from 35 to 200 MeV lying in the RBE range of 1.1–1.2. More experimental research is required to validate this result, both at VHEE and lower energy ranges. More exploration into clinical electron energies <20 MeV would also be beneficial to validate this energy independence, especially as this radiotherapy is currently used on patients. The findings from this study suggest a slightly higher RBE value for electrons than Delorme *et al.*, whose theoretical predictions found VHEE RBE to be ~1 [34].

The measurement of 1.16 ± 0.15 for proton RBE_SSB_ is within the large range of proton RBE values that have been measured over several biological endpoints, as mentioned previously. However, one similar study evaluated the response of pBR322 plasmid DNA to low-energy protons (10–30 MeV) as well as gamma rays. Vysin *et al.* [32] measured proton RBE to be between 1.01 and 1.50 for 30 MeV and 20 MeV protons, respectively, for aqueous plasmids, indicating the large range of RBE values across LET’s between 1.94 and 6.96 keV/μm. In comparison, the proton LET reached during our experiment was out of this range and significantly lower at ~0.4 keV/μm. Following Vysin *et al.*’s data set, it would suggest that our results should have an RBE closer to ~1. It would therefore be useful for more plasmid irradiation experiments to take place across varying LET’s to validate existing experiments and provide more data about how proton LET influences DNA damage in plasmid structures.

RBE_SSB_ values were measured to be between 1.1 and 1.2 for both 228 MeV protons and 35 MeV electrons, where the biological endpoint is in the pBR322 plasmid DNA, under low scavenging conditions. This result is consistent with experimental RBE values for VHEE presented in the literature, suggesting that electron RBE is energy independent. There were no significant differences between proton and electron RBE at low scavenging capacities, suggesting that these two modalities result in similar amounts of DNA damage. This study provides important and novel information on the effect of high-energy electrons on DNA, which is the first step along the way to clinical implementation of VHEE. More experimental results are required to validate both proton and electron RBE values at different beam energies, LET’s and biological endpoints. This is particularly important for VHEE and high-energy electrons, due to the minimal experimental data supporting this RBE value. Further work expanding upon plasmid DNA experiments and RBE validation through *in vitro* and *in vivo* experimentation is essential for VHEE.

## FUNDING

This work was supported by the UK Research and Innovation (UKRI), Engineering and Physical Sciences Research Council (EPSRC) [EP/T517823/1] and the UK Research and Innovation (UKRI), Science and Technology Facilities Council (STFC), Cockcroft Institute [ST/V001612/1].

## DATA AVAILABILITY

The data underlying this article is available in the article, presented in table format throughout. Any other data or specific information underlying this article will be shared on reasonable request to the corresponding author.

## CONFLICT OF INTEREST

The authors declare no conflict(s) of interest.
